# Data-Driven Parameter Selection and Modeling for Concrete Carbonation

**DOI:** 10.3390/ma15093351

**Published:** 2022-05-07

**Authors:** Kangkang Duan, Shuangyin Cao

**Affiliations:** 1 School of Civil Engineering, Southeast University, Nanjing 211189, China; 220191037@seu.edu.cn; 2 Key Laboratory of Concrete and Prestressed Concrete Structures of Ministry of Education, Southeast University, Nanjing 211189, China

**Keywords:** concrete carbonation, data mining, feature selection, machine learning, carbonation model

## Abstract

Concrete carbonation is known as a stochastic process. Its uncertainties mainly result from parameters that are not considered in prediction models. Parameter selection, therefore, is important. In this paper, based on 8204 sets of data, statistical methods and machine learning techniques were applied to choose appropriate influence factors in terms of three aspects: (1) the correlation between factors and concrete carbonation; (2) factors’ influence on the uncertainties of carbonation depth; and (3) the correlation between factors. Both single parameters and parameter groups were evaluated quantitatively. The results showed that compressive strength had the highest correlation with carbonation depth and that using the aggregate–cement ratio as the parameter significantly reduced the dispersion of carbonation depth to a low level. Machine learning models manifested that selected parameter groups had a large potential in improving the performance of models with fewer parameters. This paper also developed machine learning carbonation models and simplified them to propose a practical model. The results showed that this concise model had a high accuracy on both accelerated and natural carbonation test datasets. For natural carbonation datasets, the mean absolute error of the practical model was 1.56 mm.

## 1. Introduction

It is a well-known fact that carbonation does not normally cause damage to concrete directly [1]. However, the chemical reaction slowly destroys the alkalinity environment. CO_2_ diffuses into the concrete through interconnected pores and reacts with calcium hydroxide (CH) and hydrated calcium silicate (C-S-H) [2,3]. Consequently, it destroys the passive oxide layer of the rebar and ultimately initiates corrosion [4].

Many models, including theoretical formulas [5,6], numerical models [7,8,9], and machine learning models [10,11], have been developed over the last few decades to evaluate the carbonation status of concrete. Parameter research plays an important role in the modeling process. Previous parameter research focused on the mechanism of influence factors. Many experimental tests [12,13,14,15] have been performed to qualitatively analyze the effects that factors have on concrete carbonation and investigate the mechanism in terms of chemical reactions. 

For example, Papadakis et al. [16] suggested that replacing cement with fly ash would increase the porosity, while replacing aggregate with fly ash would decrease the porosity. Experiments in [17,18,19] suggest that replacing cement with silica fume reduces porosity since silica fume has very fine particles and a high amorphous silicon dioxide content. Large amounts of active alumina and amorphous SiO_2_ in fly ash consume the CH, but the ferrous phase in crystalline form does not participate in the pozzolanic reactions [16]. Qiang et al. [20] pointed out that steel slag had a weak reactivity. Han et al. [21] further pointed out that the main active components in steel slag were only C_2_S and C_3_S. Li et al. [22] demonstrated that the compressive strength, porosity, and permeability of concrete changed significantly during carbonation. Jiang et al. [23] explored the influence of various binder types and geometrical parameters (i.e., concrete cover thickness) on concrete carbonation and steel corrosion. The effects of supplementary cementitious materials and environmental factors such as relative humidity have been studied by many experimental tests [24,25]. 

However, present carbonation models have not involved enough work on parameter research. Some models choose parameters subjectively. For example, some empirical models take the water–cement ratio as the main parameter, while others choose concrete strength, but few studies can explain the reasons quantitatively. Currently, judging whether or not a variate can affect concrete carbonation is easy, but putting all possible factors in a model is also superfluous. Therefore, it is important to quantitatively analyze these factors.

Moreover, in terms of concrete mix design, quantitative parameter research is also necessary, since standards are needed for specifying the limitation of several indicators to control the durability of concrete. For instance, the new European Standard EN 206-1 specifies the minimum binder content and maximum water–binder ratio to guarantee the performance of concrete [26]; Chinese code GB 50010-2010 specifies the maximum water–binder ratio and the minimum strength level. 

**Figure 1:**
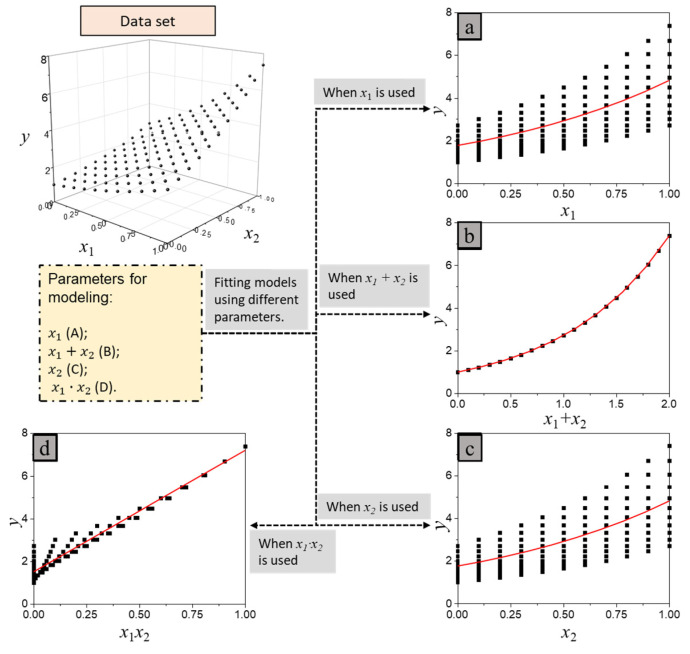
Parameters’ effects on the dispersion of Y. (**a**) *x*_1_ was used to develop a model; (**b**) *x*_1_ + *x*_2_ was used to develop a model; (**c**) *x*_2_ was used to develop a model; (**d**) *x*_1_*x*_2_ was used to develop a model.

The residual of carbonation models and the uncertainties in controlling the durability mainly result from factors that are not considered in models and concrete mix design. These uncertainties can be decreased by selecting appropriate parameters. For example, Figure 1 illustrates how parameter selection affects the residual of models. The dataset was generated by $y=e^{x_{1}+x_{2}}$. The model in Figure 1b has the best performance as it has the smallest residuals. Moreover, it is better to use *x*_1×2_ (Figure 1d) rather than *x*_1_ or *x*_2_ (Figure 1a,c) to establish a model, as the former can significantly reduce the uncertainty of the prediction. Therefore, it is important to find appropriate parameters in modeling. 

Quantitative analysis requires a large amount of test data, which is difficult to find in previous studies. In this project, many studies were consulted and a dataset including 8204 samples was established. Statistical methods were used for data-driven analysis, as well as machine learning techniques. This paper quantitatively studied influence factors in terms of three aspects: (1) the correlation between factors and concrete carbonation; (2) factors’ influence on the fluctuation of carbonation depth; and (3) the correlation between factors, which reflects the redundancy [27]. A total of 29 material-related parameters were involved. Then, we selected some parameter groups in terms of these three aspects and developed several machine learning models to verify the effectiveness. After that, one machine learning model involving a few factors was established and a practical model was proposed by simplifying it. The effectiveness of the practical model was verified via accelerated and natural carbonation datasets.

## 2. Data-Driven Parameter Selection

### 2.1. Data Collection and Description

It is necessary first to provide a concise introduction to the dataset. To make the results more reliable, 8204 sets of data, including 161 papers in Web of Science, CNKI, and WAN FANG DATA, were collected in this paper, as shown in Table 1. All concrete samples used in this study were cured for 28 days before the accelerated carbonation tests, and carbonation depth was determined by phenolphthalein. The experimental environment conditions of all the referred studies were constant. Generally, empirical models based on accelerated carbonation datasets are not appropriate for predicting actual concrete carbonation as they are often obtained under a high CO_2_ concentration. However, in terms of the relationship between factors and carbonation depth, accelerated carbonation tests are still appropriate. It is noted that the degree of the correlation is important in this part, regardless of whether this influence is positive or negative for carbonation. This implies that short-term accelerated carbonation datasets can work in this research, as some parameters such as fly ash develop a significant proportion of concrete’s strength and durability after 28 days. Parameters $p_{i}\;\left(i=C,\;S,\;A,\;F,\;\mathrm{or}\;\bar{S},\;i.e.,\;\mathrm{CaO},\;{\mathrm{SiO}}_{2},\;{\mathrm{Al}}_{2}O_{3},\;\;{\mathrm{Fe}}_{2}O_{3},\;\mathrm{or}\;{\mathrm{SO}}_{3}\right)$ can be calculated by:

(1) $$p_{i}=\;\sum p_{i,k}\cdot p_{k}+\;\sum p_{i,Cemj}\cdot p_{Cemj}$$

where $p_{i}$ is the weight of i used per unit volume of concrete; $p_{k}$ is the weight of material k (k=FA,FS,SA, or SS, i.e., fly ash, furnace slag, silica ash, or steel slag) used per unit volume of concrete; $p_{i,k}$ is the content of i in material k; $p_{Cemj}$ is the weight of cement of class j; $p_{i,Cemj}$ is the content of i in class j cement, as is explained in Table 1. In addition, furnace slag and fly ash were classified by their fineness and specific surface area according to ground granulated blast-furnace slag used for cement, mortar, and concrete GB/T 18046-2017 and fly ash used for cement and concrete GB/T 1596-2017, respectively. Table 1 exhibits detailed information about the dataset.

### 2.2. Parameter Evaluation and Selection

Parameter selection is always a hot topic in data science, and it has recently been applied in civil engineering. Some studies [28,29] adopted conventional parameter selection methods for solving energy issues in buildings. Conventional selection methods aim to remove irrelevant and redundant information from the dataset according to two criteria: the correlation between the parameters and the object (i.e., remove irrelevant information), and the correlation between the parameters (i.e., find redundant information) [30]. 

Correlation analysis methods mainly include Pearson’s correlation coefficient, Spearman’s correlation coefficient, Kendall’s correlation coefficient, maximal information coefficient (MIC) [31,32], etc. Selection methods based on them are called filter methods [33]. In addition, wrappers and embedded methods are also widely used for selection [27]. Decision trees, naive Bayes, and support vector machines [34,35] are several popular predictors. For these predictors, the criterion essentially depends on the loss function of the predictors. The selection of approaches for correlation analyses is based on the statistical characteristics of data and the target of the study. 

In terms of statistics, the dataset used in this study was not appropriate for some methods, which require the dataset to comply with the Gaussian distribution. In addition, some new attributes should be included:

- Parameter performance in controlling and predicting the durability of concrete under the impact of uncertainties of carbonation depth needs to be evaluated.
- Parameters should reduce the dispersion of carbonation depth.

#### 2.2.1. Method

Except for the correlation analysis, a quantitative analysis of a parameter’s effects on the dispersion of carbonation depth is needed. 

For the first aspect, $CORR_{k}$ was used to denote the correlation between parameter k and carbonation depth. A high $CORR_{k}$ represented a strong correlation. Spearman’s correlation coefficient, Pearson’s correlation coefficient, and Kendall’s correlation coefficient are common correlation analysis indices. Spearman’s correlation coefficient and Kendall’s correlation coefficient are copula-based random variable dependency measurement indices. Compared with Pearson’s correlation coefficient, they do not require that datasets conform to a special distribution. Generally, Spearman’s correlation coefficient is the Pearson correlation coefficient calculated from the vectors of ranks [36]. The Pearson correlation coefficient of vectors *X* and *Y* can be calculated by [33]:

(2) $$\rho \left(X,Y\right)=\frac{1}{N-1}\sum_{i=1}^{N}\left(\frac{X_{i}-\bar{X}}{\sqrt{\frac{1}{\left(N-1\right)}\sum_{j=1}^{N}{\left(X_{j}-\bar{X}\right)}^{2}}}\right)\left(\frac{Y_{i}-\bar{Y}}{\sqrt{\frac{1}{\left(N-1\right)}\sum_{j=1}^{N}{\left(Y_{j}-\bar{Y}\right)}^{2}}}\right)$$

where N is the number of samples and $\bar{X}$ is the average value. Then, the Spearman correlation coefficient of vectors X and Y can be calculated by replacing the values in each vector with their ranks. 

For the second aspect, $VARR_{k}$ depicted k’s effect on the dispersion of carbonation depth. The uncertainties of an observed value such as carbonation depth are usually described by its variance. A high variance means a large dispersion; predicting the carbonation depth with its average value is thus unreliable. Therefore, $VARR_{k}$ was defined as the reduction degree of uncertainties of the observed carbonation depth data after parameter k was used, which can be written as:

(3) $$VARR_{k}=\frac{Var-{\tilde{Var}}_{k}}{Var}$$

where Var represents the standard deviation of carbonation depth x when no parameter is considered. If the usage of parameter k does not affect the dispersion of carbonation depth, Var will be equal to ${\tilde{Var}}_{k}$ and VARR will be equal to zero. Var can be obtained by Equation (4):

(4) $$Var=\sqrt{\frac{1}{\left(N-1\right)}\sum_{i=1}^{N}{\left(X_{i}-\bar{X}\right)}^{2}}$$

where N is the number of samples in the original dataset D, $X_{i}$ is the carbonation depth of the ith sample, and $\bar{X}$ is the average value. The uncertainties of x after adopting parameter k can be obtained by Equation (5):

(5) $${\tilde{Var}}_{k}=\sum_{i=1}^{t}\frac{N_{i}}{N}{\tilde{Var}}_{i,k}$$

where ${\tilde{Var}}_{i,k}$ denotes the standard deviation of x at $k=k_{i}$ and $k_{i}$ is one of the values of k.$\;N_{i}$ is the number of samples at $k=k_{i}$. Assume the dataset including $N_{i}$ samples named $D_{i}$, and $N_{i}/N$ represents the weight of $D_{i}$. Suppose that k has t values $\left(k=k_{1},\;k_{2},\;\ldots ,k_{t}\right)$, and ${\tilde{Var}}_{i,k}$ can be calculated by Equation (6):

(6) $${\tilde{Var}}_{i,k}=\sqrt{\frac{1}{\left(N-1\right)}\sum_{j=1}^{N_{i}}{\left(X_{ij}-{\bar{X}}_{i}\right)}^{2}},\;\;\left(i=1,\ldots ,t\right)$$

where $X_{ij}$ is the carbonation depth of the jth sample in $D_{i}$ and ${\bar{X}}_{i}$ is the mean of x in $D_{i}$. According to Equation (6), if k does not affect the distribution of carbonation depth, D and $D_{i}$ have the same distribution (i.e., $Var={\tilde{Var}}_{i,k}$). This can be verified by linking an irrelevant variate $k^{\prime }$ produced by random sampling to carbonation depth, and calculating the value of ${\tilde{Var}}_{i,k^{\prime }}$. Therefore, the importance of parameter k can be evaluated by Equation (7):

(7) $$I_{k}=\frac{VARR_{k}}{\max VARR}\cdot \frac{CORR_{k}}{\max CORR}$$

where $I_{k}$ is the importance coefficient of parameter k, maxVARR is the max value of VARR, and maxCORR is the max value of CORR. As shown in Equation (7), parameters that have a weak correlation with carbonation or that do not reduce the uncertainties will make $I_{k}$ be equal to zero. 

In addition, it is noted that there is still one possible issue in the calculation of ${\tilde{Var}}_{i,k}$: ${\tilde{Var}}_{i,k}$’s sensitivity to the outlier. This was handled by identifying and excluding outliers. IQR is a common index for finding outliers in statistics. *IQR* can be calculated by:

(8) $$IQR=Q_{3}-Q_{1}$$

where $Q_{1}$ is the 25th percentile and $Q_{3}$ is the 75th percentile. Moreover, x is considered an outlier if it meets:

(9) $$x\langle \;Q_{1}-1.5\left(IQR\right)\;or\;x\rangle \;Q_{3}+1.5\left(IQR\right)$$

**Figure 2:**
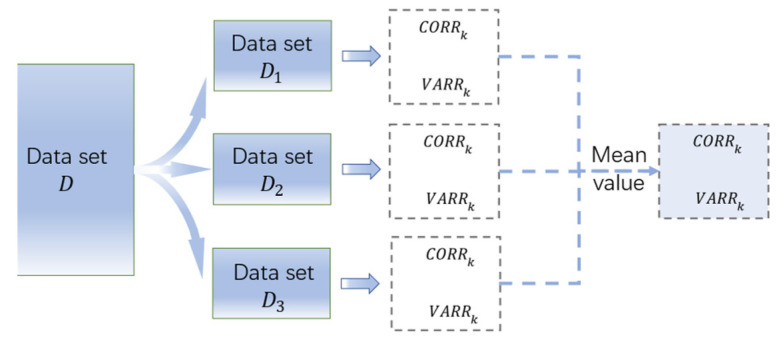
Splitting of dataset *D* and calculation.

Deleting outliers changes the dataset used for calculating $VARR_{k}$. To maintain the consistency of the dataset, the calculation of $CORR_{k}$ and $VARR_{k}$ used the same dataset. In addition, to improve the stability of evaluation results, the original dataset, D, was split into three child datasets $D_{l}\;\left(l=1,\;2,\;3\right)$. Simple random sampling without replacement was used for splitting. For dataset $D_{l}$, about 2000~2600 sets of valid data were included in it. The final results of the evaluation are the mean of the results of the three child datasets, as shown in Figure 2.

#### 2.2.2. Results and Discussions

**Figure 3:**
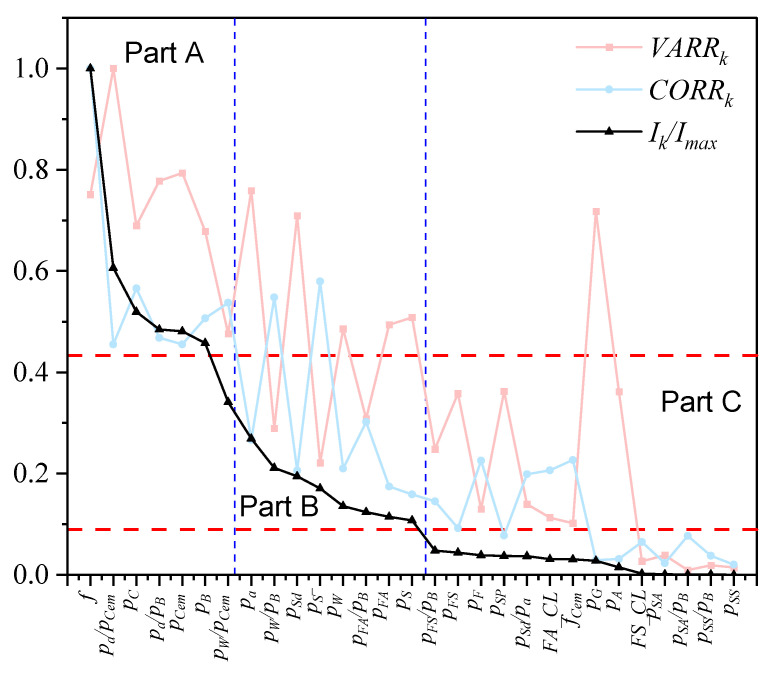
Analysis results of each factor.

Based on the above discussions, for parameter k, its $CORR_{k}$, $VARR_{k}$ and $I_{k}$ can be calculated, as shown in Figure 3. It is noted that these parameters can be divided into three parts (Parts A, B, and C) by the blue and red dash lines according to their $I_{k}$. The compressive strength *f* was most important in comparison with other factors. In addition, Figure 3 exhibits the $CORR_{k}$ and $VARR_{k}$ of the parameters. The compressive strength *f* had the highest $CORR_{k}$ and a high $VARR_{k}$, which implied that its correlation with carbonation depth was the highest and that it could also largely reduce the uncertainty of carbonation depth. The aggregate–cement ratio $p_{a}/p_{Cem}$ had the highest $VARR_{k}$ and could thus reduce the uncertainty to the lowest value, while it only had a medium $CORR_{k}$. 

The parameters in Part A had a high $I_{k}$ and also had a high $CORR_{k}$ and $VARR_{k}$ (Figure 3). In Part B, parameters that had a high $CORR_{k}$ usually had a low $VARR_{k}$, which implied that indices $CORR_{k}$ and $VARR_{k}$ revealed different aspects of the influence of the factors.

In terms of mechanism, it is easy to understand that the compressive strength *f* had the highest $CORR_{k}$. For example, Papadakis et al. [37] proposed a function to calculate the compressive strength of fly ash concrete in terms of chemical reactions:

(10) $$f=38.8\left[\frac{p_{Cem}+\frac{3p_{FA}}{2p_{B}}\left(1-\frac{0.5p_{W}}{p_{Cem}}\right)}{p_{W}}-0.5\right]\;$$

Equation (10) shows that the water content $p_{W}$ is negative in relation to the concrete strength *f*. Moreover, $p_{W}$ is negative in relation to carbonation depth. Further, it is believed that the effects of $p_{W}$ on porosity are also the main reason for its influence on *f*. In addition, Equation (10) also demonstrates that replacing cement with fly ash (assume $p_{B}$ is a constant) causes a low strength, but replacing the aggregate with fly ash ($p_{B}$ is not a constant) increases the strength. This might not comply with some situations as fly ash mainly affects the early strength. Fly ash and other supplementary cementitious materials also have a similar correlation with carbonation depth. In sum, compressive strength *f* shows a strong uniformity with carbonation depth x. 

The results also showed that $p_{a}/p_{Cem}$ had the highest $VARR_{k}$. The performance of the aggregate–cement ratio $p_{a}/p_{Cem}$ in reducing the uncertainty of carbonation depth mainly resulted from two aspects: its influence on the distribution of carbonation depth and the number of its values. However, only increasing the number of values cannot reduce dispersion. 

## 3. Parameter Combinations and Verification

### 3.1. Evaluation of Factor Groups

This part aims to provide some combinations for developing prediction models and concrete mix design. Although Section 2 provides the evaluation results of single factors, combinations should make a further selection, since some parameters have high $I_{k}$ simply because they share the same attribute. For example, CaO content, cement content, and binder content all have high $I_{k}$ since they all reflect the content of reactants such as Ca(OH)_2_; i.e., they are interrelated. Groups containing interrelated parameters will have much redundant information [27]. Table 2 provides a reference for evaluating the relationship between parameters.

As the correlation between factors reflects the possibility that one factor can be replaced by others and $I_{k}$ denotes the importance, the performance of different factor groups can be estimated through the following steps:

1. Determine the number of parameters included in the group;
2. Assume that one group consists of *m* factors, sort all factors from largest to smallest according to their $I_{k}$, and calculate $S_{m}$;
  (11) $$S_{m}=\sum_{i=1}^{m}R_{i}\cdot I_{i}\;$$
  where $R_{i}$ implies the possibility that factor i cannot be replaced by previous factors and $I_{i}$ denotes the $I_{k}$ of factor i. $R_{i}$ can be calculated by:
  (12) $$R_{i}=\sum_{j=1}^{i-1}R_{j}\cdot \left(1-\left|r_{i,j}\right|\right)\cdot H\;$$
  where $r_{i,j}$ denotes Spearman’s correlation coefficient between i and j, and H is used to make sure that $1-\left|r_{i,j}\right|\geq 0$; i.e., if $1<\left|r_{i,j}\right|$, H=0; otherwise, H=1. It is noted that $R_{1}$ should be equal to one.
3. Sort all groups from largest to smallest according to their $S_{m}$.

As shown in Table 3, several combinations were provided and groups containing the same number of factors have similar $S_{m}$. In the next part, this paper discusses the effectiveness of these combinations via machine learning methods.

### 3.2. Validation of Suggested Parameters

To verify the validity of the parameter combinations listed in Table 3, machine learning (ML) methods were used in this section. It is noted that environmental factors such as temperature were also included in machine learning models. 

To improve the reliability, three ML approaches were used: support vector regression (SVR), XGboost, and deep neural networks (DNN). Current ML techniques combine datasets and algorithms to find the relationship between parameters and the target. Once appropriate parameters are selected, models usually have very a high accuracy [38]. Therefore, ML models were used in this study to investigate whether or not the suggested combinations had significant advantages in predicting carbonation depth. Considering that readers might be unfamiliar with these ML methods, some brief introductions were given.

#### 3.2.1. ML Methods

Conventional regression or fitting algorithms such as linear regression first assume a formula with undetermined coefficients. For example, $x^{\prime }=w\cdot t+b$. Then, this approach uses optimization algorithms to adjust the values of undetermined coefficients (w and b) to reduce the error between the true carbonation depth x and the predicted value $x^{\prime }$ (e.g., minimize $loss={\left(x-x^{\prime }\right)}^{2}$) to the minimum. These methods generate linear models. For complex issues, researchers need to guess the basic form of fitted curves. 

SVR uses the kernel function $K\left(t_{1}\cdot t_{2}\right)=\phi \left(t_{1}\right)\cdot \phi \left(t_{2}\right)$ to convert a low-dimensional space into a high-dimensional space [38]. Therefore, nonlinear curves in the low-dimensional space can be fitted by a hyperplane. In addition, the loss function SVR used has high robustness due to the addition of relaxation coefficient ϵ. If the gap between x and $x^{\prime }$ is lower than ϵ, the error is ignored. Detailed discussions and tutorials of SVR can be found in [39]. The expression can be written as:

(13) $$x^{\prime }=w\cdot \phi \left(t\right)+b\;$$

where $w=\left(\left[\hat{a}\right]-\left[a\right]\right)\cdot \phi \left(\hat{t}\right)$. $\hat{t}$ is the vector of the parameters of the dataset; $\left[\hat{a}\right]$ and [a] are the diagonal matrices of undetermined coefficients. $\phi \left(\hat{t}\right)\cdot \phi \left(t\right)$ denotes the kernel function. In this study, the radial basis kernel function $K\left(t_{1}\cdot t_{2}\right)=e^{-\gamma {\left(t_{1}-t_{2}\right)}^{2}}$ was used. For the widely noted overfitting problem in ML models, SVR also has one important characteristic: $\left\|w^{2}\right\|$ in its regularized risk function can help it avoid overfitting.

Different from SVR, XGboost is one kind of boosting algorithm [40]. XGboost first develops a weak regression model. For example, in this paper, the regression tree model $F_{0}\left(t\right)$ was used. Then, the next weak model $F_{1}\left(t\right)$ is built to reduce the error between x and $x^{\prime }$ of the tree model. This step cycles many times, and for the mth time, the predicted carbonation depth $x^{\prime }$ is:

(14) $$x_{m}^{\prime }\left(t\right)=x_{m-1}^{\prime }\left(t\right)+\alpha_{m}F_{m}\left(t\right)$$

Mean square error (MSE) was used to evaluate the error between x and $x^{\prime }$, and MSE can be calculated by:

(15) $$MSE\left(x,x^{\prime }\right)=\frac{1}{N}\sum_{i=0}^{N-1}{\left(x_{i}-x_{i}^{\prime }\right)}^{2}$$

To avoid overfitting, the regularization item L2 was used in the training of the XGboost models. Detailed information on XGboost is listed in [40].

**Figure 4:**
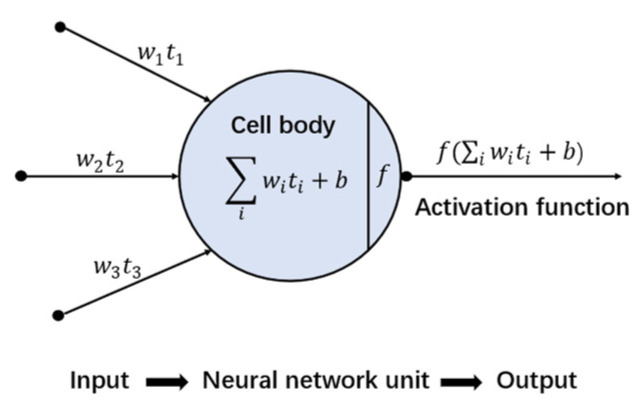
An illustration of a neuron.

**Figure 5:**
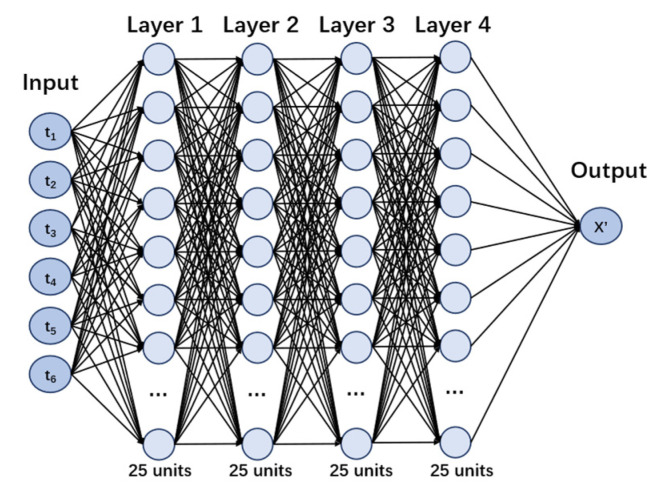
An illustration of a deep neural network.

DNN is similar to the human nervous system, composed of many neurons. Those neurons are stored in different layers and neurons in different layers are connected. Every neuron in the neural network receives signals from neurons that are linked to it. Processed by the activation function in the neuron, signals are passed to later neurons, as shown in Figure 4. The hyperbolic tangent activation function was used in this study. DNN can simulate the most complex function due to the combination of many neurons and the process of the activation function [41]. Figure 5 shows the structure of a DNN with six parameters. To avoid overfitting, the dropout approach was used [42]. Dropout randomly makes some neurons invalid at a probability of p during each period of training, and all neurons are used for final models. Of course, to maintain the scale of the predicted value, the weight of the neurons in the final models will multiply p. In this study, p=0.3, meaning that, for each layer, 30% of the neurons were randomly set as invalid during each period of training. In addition, the regularization item L2 was also used to avoid overfitting.

#### 3.2.2. Verification and Discussions

**Figure 6:**
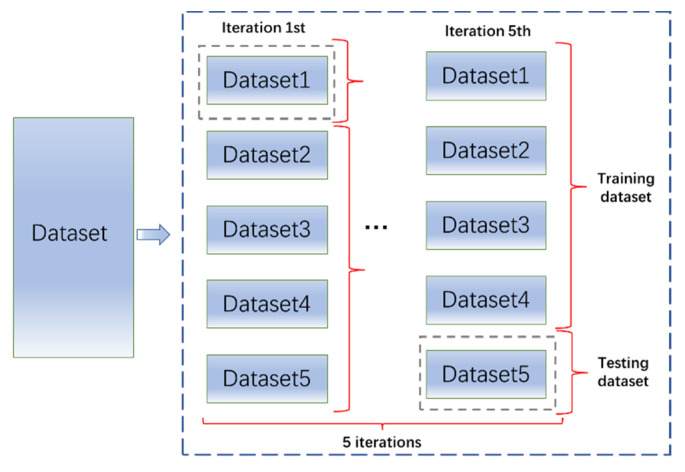
Illustration of 5-fold cross-validation.

In this part, 1825 sets of data were used for verification. For each group, ML models were built to compute the MSE of given combinations. Combinations with low MSE were believed to be effective. To improve the stability, five-fold cross-validation was used. The dataset was evenly divided into five subsets. Four subsets were used to train a model and one subset was used to test the model. This process cycles five times and uses different subsets as the testing data each time. The final results were based on the mean results of five models, as is shown in Figure 6. In addition, data normalization was used for preprocessing. Table 4 shows the MSE results of the models. As is shown in Table 4, Group 17-1 used all parameters. Parameters in Group 5-3 reflected the mix design of concrete. 

For groups listed in Table 3, results showed that the MSE of models decreased with the addition of new parameters. However, with the appending of new parameters, this effect declined. Compared with Group 17-1, Groups 5-1 and 5-2 had similar MSEs. In this study, five parameters can finely approximate the performance of all parameters. Furthermore, as using too many parameters would increase the complexity of the models, Group 5-2 showed a better accuracy than Group 17-1. Compared with Group 5-3, which reflected the mix design of concrete, Groups 3-1~3-3 showed a similar accuracy. All ML models showed that the method proposed in this paper can choose appropriate parameters and reduce the demand for the number of parameters.

## 4. Practical Carbonation Models for Existing Concrete Structures

In previous sections, factor groups were proposed and several ML models were developed. For existing concrete structures, it is very difficult to obtain parameters, as some original design information may be unavailable. According to Section 2 and Section 3, this paper developed a prediction model containing necessary environmental factors and two concrete-related factors (compressive strength and aggregate–cement ratio) via neural network methods. 

**Figure 7:**
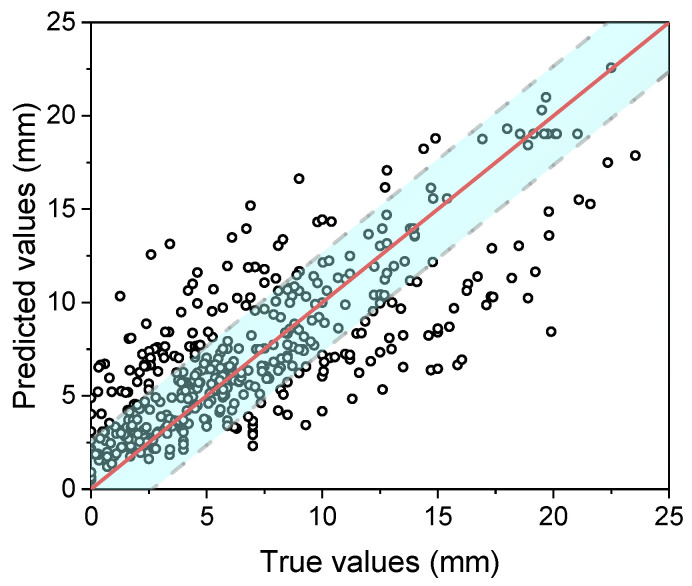
Illustration of 5-fold cross-validation.

Neural network methods and the settings of the models were discussed in Section 3.2. This model included six input parameters (humidity, temperature, the concentration of CO_2_, compressive strength, aggregate–cement ratio, and time), four hidden layers containing 25 units in each layer, and one output value (Figure 5). The dataset used for training and testing was the dataset described in Table 1. A total of 90% of the dataset was used for training and 10% was used for testing the model. It is noted that the concentration of CO_2_ ranged from 1% to 50%, the temperature ranged from 10 °C to 60 °C, the relative humidity ranged from 35% to 95%, and the carbonation time ranged from 1 day to 364 days. The results are shown in Figure 7. Most points were located in the blue area (±2.65 mm), and the mean error was 2.5 mm. It is not necessary to compare ML models with other empirical models or theoretical models, as ML models always perform better on the testing dataset, as has been demonstrated by many studies [43,44]. 

To make the model more convenient, this part also aims to propose a practical model for the carbonation prediction of existing structures based on the ML model.

### 4.1. Establishment of the Practical Model

**Figure 8:**
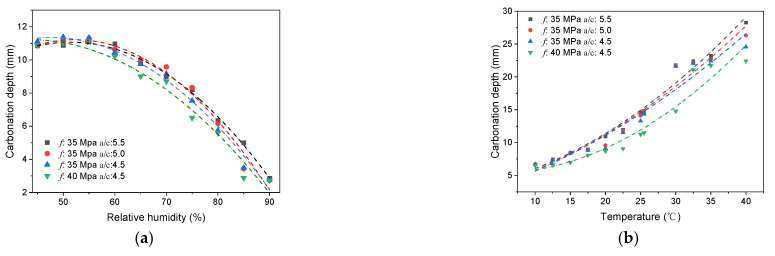

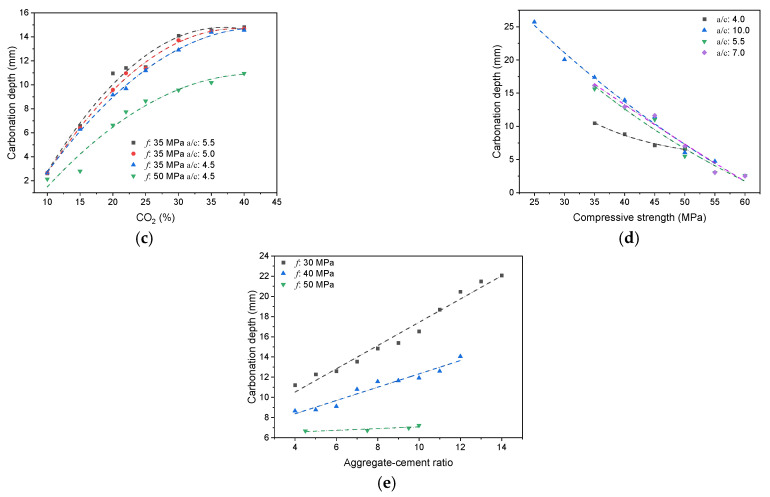
Relationship between carbonation depth and factors contained in the ML model: (**a**) the relationship between carbonation depth and relative humidity; (**b**) the relationship between carbonation depth and temperature; (**c**) the relationship between carbonation depth and CO_2_ concentration; (**d**) the relationship between carbonation depth and compressive strength; (**e**) the relationship between carbonation depth and aggregate–cement ratio.

According to the ML model, six parameters were used to predict the carbonation depth; the function of the practical model can thus be written as:

(16) $$x=f_{1}\left(RH\right)f_{2}\left(T\right)f_{3}\left(\left[{\mathrm{CO}}_{2}\right]\right)f_{4}\left(f,a/c\right)\sqrt{t}$$

where RH is the relative humidity, T denotes the temperature, $\left[{\mathrm{CO}}_{2}\right]$ is the concentration of CO_2_, f is the compressive strength, a/c denotes the aggregate–cement ratio, and t is the carbonation time. A dataset was created to explore the relationship between carbonation depth and parameters contained in the ML model. The results are shown in Figure 8.

Figure 8a shows the influence of relative humidity, where *f* denotes the compressive strength and *a*/*c* is the aggregate–cement ratio. The influence of the relative humidity had a peak value for two reasons: (1) carbonation reactions occurred in the pore solution, and water was thus needed; (2) water can form water films on the pore surface and then impede the diffusion of CO_2_ in pores. Since the data whose relative humidity was below 50% was insufficient in comparison with the data whose relative humidity was greater than 50%, only samples whose relative humidity was greater than 50% were involved, and $f_{1}\left(RH\right)$ can be assumed to be linear with an insignificant loss of accuracy. This also largely simplifies the calculation.

Figure 8b shows the influence of temperature on carbonation. High temperatures can raise the rate of diffusion of CO_2_ gas and accelerate chemical reactions. Arrhenius formulas are usually used to depict the temperature’s effects on chemical reactions. By the same token, a linear function was assumed to simplify the calculation and the approximate temperature’s effects on carbonation with an insignificant loss of accuracy. 

Figure 8c shows the influence of CO_2_ concentration on carbonation. In the early stages of carbonation, with the increase in CO_2_ concentration, the carbonation reaction rate increases. However, CaCO_3_ generated by CO_2_ concentrations that are too high will fill pores and impede the contact between Ca(OH)_2_ and CO_2_, which finally hinders carbonation. $f_{3}\left(\left[{\mathrm{CO}}_{2}\right]\right)$ was assumed to be a square root function.

Figure 8d shows the relationship between concrete strength and carbonation depth. The influence of compressive strength has been widely discussed in many studies, and a power function is usually used. Figure 8e shows that the influence of the aggregate–cement ratio can be approximated as a linear function. Considering that both the aggregate–cement ratio and the concrete strength are concrete-related parameters, the relationship between them in $f_{4}\left(f,a/c\right)$ can be further divided into the linear combination form ($Ag_{1}\left(f\right)+Bg_{2}\left(a/c\right)+C$) or the product form ($g_{1}\left(f\right)g_{2}\left(a/c\right)$). Figure 8e shows that with the increase in concrete strength, the effects of the aggregate–cement ratio on carbonation are weakened, which indicates that $g_{1}\left(f\right)g_{2}\left(a/c\right)$ can better depict this change. 

The testing dataset and the training dataset used to determine the values of the coefficients were split from the same dataset given in Table 1, and the testing dataset constituted 10% of the total dataset. Finally, the function can be written as:

(17) $$\{\begin{array}{c} f_{1}\left(RH\right)=\left(-0.49RH+0.49\right)\\ f_{2}\left(T\right)=0.26T-0.02\\ f_{3}\left(\left[{\mathrm{CO}}_{2}\right]\right)=0.275\sqrt{\left[{\mathrm{CO}}_{2}\right]}\\ f_{4}\left(f,\frac{a}{c}\right)=\left(\frac{24.48}{\sqrt{f}}-2.74\right)\left(\frac{0.04a}{c}+1.26\right) \end{array}$$

### 4.2. Verification of Models

To verify the accuracy of the practical model, the testing dataset was used. Other carbonation models [6,45,46,47,48] were also applied to the testing dataset, as shown in Table 5. This practical model had lower errors in comparison with other models. Monteiro’s model [45], Niu’s model [47], and this practical model take concrete strength as the main parameter, which showed significant advantages in accuracy compared with other models and also corroborated the results in Section 2.

The median value of the errors is also listed in Table 5. Since the median value is not affected by outlier samples, it can better reflect the actual performance of the models. The median value was higher than the mean value, which means that there were some outlier experimental results in the dataset. Table 5 also shows that this practical model exhibited a better accuracy at each stage of the carbonation process.

To further verify the effectiveness of this model, this paper collected an extra natural carbonation dataset [49,50,51,52] to explore its accuracy. This dataset included 76 sets of data. The natural carbonation time ranged from 28 to 9125 days, and the locations involved the northern and southern regions as well as the central and western regions of China. It is noted that the curing conditions of the concrete specimens used in natural carbonation tests were not standard curing. Some of the specimens were placed in an indoor environment and others were placed in an outdoor environment. Previous studies suggested the carbonation depth should be multiplied by the coefficients of 2.81 and 1.50 for specimens in the indoor and the outdoor environment, respectively. Table 6 shows the final results. The mean absolute error was 1.56 mm; the practical model thus had a high accuracy.

## 5. Conclusions

This study aimed to explore a way of evaluating and selecting the most effective factors for developing concrete carbonation models and controlling the durability of concrete through big data. Statistical analyses and ML techniques were used in this study. The following conclusions can be drawn:

1. Single-parameter analysis showed that compressive strength had the highest correlation with carbonation depth and that the aggregate–cement ratio can minimize the uncertainties of carbonation depth. The method proposed in this study to evaluate single factors and the effects of parameter groups was effective. The results showed that the mean square error of ML models using the three selected parameters can reach 14.04 mm^2^, which was lower than the values obtained with ML models using six concrete mix design parameters (17.67 mm^2^) and close to the results from ML models using all parameters (11.61 mm^2^). Because appropriate groups improved the models’ performance and reduced their complexity, the mean square error of ML models using five selected parameters was able to reach 11.01 mm^2^, which was even lower than the values obtained with ML models using all parameters.
2. Several machine learning models were developed to predict concrete carbonation depth. Through appropriate parameter selection, the models realized a high accuracy with a few parameters. For existing concrete structures, two concrete-related parameters (concrete strength and aggregate–cement ratio) and environmental factors were used to build a model via neural network methods. The results showed that the mean error of this model was about 2.5 mm. Based on this model, an empirical model was developed. The model was very simple and calculation-friendly. The results showed that this practical model had a high accuracy on both accelerated and natural carbonation datasets (mean absolute error = 1.56 mm).

## Figures and Tables

**Table 1:** Details of the dataset.

Factors	Unit	Histogram	Min	Max	Mean	Valid	Explanation
$p_{Cem}$	kg/m^3^	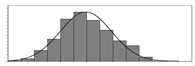	30	693	297	7351	Weight of cement used per unit volume of concrete
$p_{W}$	kg/m^3^	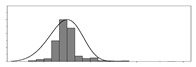	80	456	176	7351	Weight of water used per unit volume of concrete
$p_{FA}$	kg/m^3^	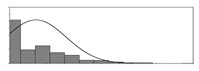	0	506	72	7351	Weight of fly ash used per unit volume of concrete
$p_{FS}$	kg/m^3^	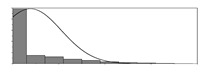	0	372	39	7351	Weight of furnace slag used per unit volume of concrete
$p_{SS}$	kg/m^3^	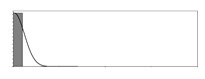	0	228	1	7351	Weight of steel slag used per unit volume of concrete
$p_{SA}$	kg/m^3^	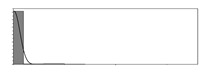	0	59	1	7351	Weight of silica ash used per unit volume of concrete
$p_{Sd}$	kg/m^3^	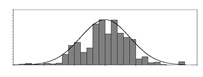	367	1071	700	6573	Weight of sand used per unit volume of concrete
$p_{G}$	kg/m^3^	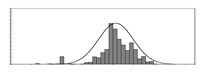	601	1319	1114	6573	Weight of gravel used per unit volume of concrete
$p_{SP}$	-	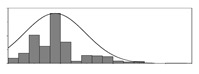	0	3.5	0.9	6695	Weight of water reducer used per unit weight of the binder
$f_{Cem}$	Mpa	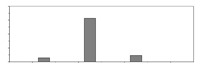	32.5/42.5/52.5			7787	Cement strength level
FA_CL	-	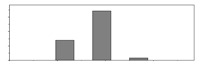	Ⅰ/Ⅱ/Ⅲ			6491	Class of fly ash
FS_CL	-	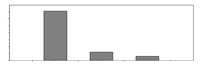	S75/S95/S105			3924	Class of furnace slag
$p_{a}$	kg/m^3^	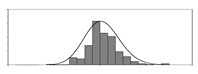	970	2237	1815	6573	Weight of aggregate used per unit volume of concrete
$p_{B}$	kg/m^3^	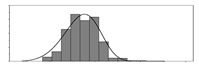	200	842	409	7351	Weight of binder used per unit volume of concrete
$p_{W}/p_{Cem}$	-	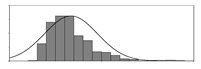	0.25	4	0.67	8100	Water–cement ratio
$p_{W}/p_{B}$	-	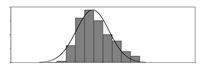	0.23	0.95	0.44	8100	Water–binder ratio
$p_{Sd}/p_{a}$	-	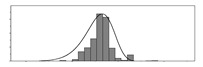	0.23	0.56	0.39	7308	Sand ratio
$p_{a}/p_{Cem}$	-	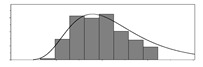	2.23	70	7.14	6573	Aggregate–cement ratio
$p_{a}/p_{B}$	-	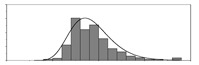	1.86	10.5	4.66	6573	Aggregate–binder ratio
$p_{FA}/p_{B}$	%	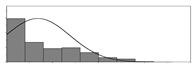	0	80	17	8133	Percentage of fly ash
$p_{FS}/p_{B}$	%	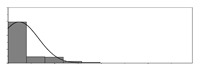	0	80	16	8133	Percentage of furnace slag
$p_{SS}/p_{B}$	%	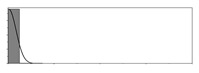	0	60	0.3	8133	Percentage of steel slag
$p_{SA}/p_{B}$	%	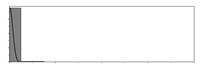	0	15	0.2	8133	Percentage of silica ash
$p_{C}$	kg/m^3^	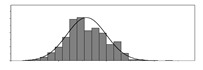	56	436	206	7289	Weight of CaO used per unit volume of concrete
$p_{S}$	kg/m^3^	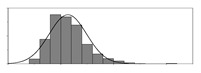	42	305	113	7289	Weight of SiO_2_ used per unit volume of concrete
$p_{A}$	kg/m^3^	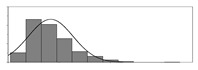	10	159	42	7289	Weight of Al_2_O_3_ used per unit volume of concrete
$p_{F}$	kg/m^3^	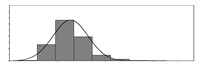	7	55	17	7289	Weight of Fe_2_O_3_ used per unit volume of concrete
$p_{\bar{S}}$	kg/m^3^	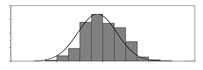	3	15	8	7289	Weight of SO_3_ used per unit volume of concrete
f	Mpa	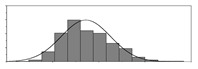	8	95	43	5781	Compressive strength of concrete at 28 days

**Table 2:** Spearman’s correlation coefficient of top parameters.

	$p_{W}/p_{B}$	$p_{B}$	$p_{W}$	$p_{Cem}$	$p_{W}/p_{Cem}$	$p_{FA}$	$p_{S}$	$p_{C}$	$p_{\bar{S}}$	f	$p_{FA}/p_{B}$	$p_{Sd}$	$p_{G}$	$p_{a}$	$p_{a}/p_{B}$	$p_{a}/p_{Cem}$
$p_{W}/p_{B}$	1	−0.69	0.47	−0.22	0.4	−0.16	−0.54	−0.35	−0.47	−0.47	−0.1	0.29	0.29	0.51	0.66	0.28
$p_{B}$	−0.69	1	0.22	0.43	−0.26	0.19	0.75	0.61	0.76	0.49	0.09	−0.5	−0.3	−0.7	−0.98	−0.52
$p_{W}$	0.47	0.22	1	0.19	0.22	0.03	0.15	0.21	0.23	−0.15	−0.01	−0.18	−0.05	−0.19	−0.21	−0.21
$p_{Cem}$	−0.22	0.43	0.19	1	−0.87	−0.44	−0.15	0.89	0.84	0.25	−0.5	−0.33	0.01	−0.27	−0.44	−0.99
$p_{W}/p_{Cem}$	0.4	−0.26	0.22	−0.87	1	0.46	0.28	−0.73	−0.66	−0.3	0.49	0.23	0	0.2	0.28	0.86
$p_{FA}$	−0.16	0.19	0.03	−0.44	0.46	1	0.69	−0.53	−0.32	−0.05	0.99	−0.03	−0.24	−0.25	−0.18	0.37
$p_{S}$	−0.54	0.75	0.15	−0.15	0.28	0.69	1	0	0.22	0.32	0.61	−0.33	−0.34	−0.58	−0.74	0.07
$p_{C}$	−0.35	0.61	0.21	0.89	−0.73	−0.53	0	1	0.95	0.39	−0.61	−0.36	−0.05	−0.33	−0.59	−0.89
$p_{\bar{S}}$	−0.47	0.76	0.23	0.84	−0.66	−0.32	0.22	0.95	1	0.42	−0.42	−0.42	−0.14	−0.46	−0.74	−0.86
f	−0.47	0.49	−0.15	0.25	−0.3	−0.05	0.32	0.39	0.42	1	−0.11	−0.2	0	−0.2	−0.42	−0.27
$p_{FA}/p_{B}$	−0.1	0.09	−0.01	−0.5	0.49	0.99	0.61	−0.61	−0.42	−0.11	1	0.01	−0.2	−0.18	−0.08	0.44
$p_{Sd}$	0.29	−0.5	−0.18	−0.33	0.23	−0.03	−0.33	−0.36	−0.42	−0.2	0.01	1	−0.22	0.65	0.56	0.4
$p_{G}$	0.29	−0.3	−0.05	0.01	0	−0.24	−0.34	−0.05	−0.14	0	−0.2	−0.22	1	0.51	0.36	0.06
$p_{a}$	0.51	−0.7	−0.19	−0.27	0.2	−0.25	−0.58	−0.33	−0.46	−0.2	−0.18	0.65	0.51	1	0.8	0.39
$p_{a}/p_{B}$	0.66	−0.98	−0.21	−0.44	0.28	−0.18	−0.74	−0.59	−0.74	−0.42	−0.08	0.56	0.36	0.8	1	0.52
$p_{a}/p_{Cem}$	0.28	−0.52	−0.21	−0.99	0.86	0.37	0.07	−0.89	−0.86	−0.27	0.44	0.4	0.06	0.39	0.52	1

**Table 3:** Parameter groups.

Number of Factors	No.	Groups
3 factors	3-1	$f,\;p_{a}/p_{Cem}$ $,\;p_{W}$
	3-2	$f,\;p_{a}/p_{Cem}$ $,\;p_{Sd}$
	3-3	$f,\;p_{a}/p_{Cem}$ $,\;p_{a}$
4 factors	4-1	$f,\;p_{a}/p_{Cem}$ $,\;p_{a}$ $,\;p_{w}$
	4-2	$f,\;p_{a}/p_{Cem}$ $,\;p_{a}$ $,\;p_{FA}$
	4-3	$f,\;p_{a}/p_{Cem}$ $,\;p_{a}$ $,\;p_{FA}/p_{B}$
5 factors	5-1	$f,\;p_{a}/p_{Cem}$ $,\;p_{a}$ $,\;p_{w}$ $,\;p_{FA}$
	5-2	$f,\;p_{a}/p_{Cem}$ $,\;p_{a}$ $,\;p_{w}$ $,\;p_{Sd}$

**Table 4:** Verification of combinations.

No.	Groups	SVR-MSE	XGB-MSE	DNN-MSE
3-1	$[{\mathrm{CO}}_{2}],RH,\;T,\;t,\;f,\;p_{a}/p_{Cem}$ $,\;p_{W}$	21.62	14.04	18.68
3-2	$[{\mathrm{CO}}_{2}],RH,\;T,t,\;f,\;p_{a}/p_{Cem}$ $,\;p_{Sd}$	22.64	15.46	17.85
3-3	$[{\mathrm{CO}}_{2}],RH,\;T,\;t,\;f,\;p_{a}/p_{Cem}$ $,\;p_{a}$	20.96	14.20	16.62
4-1	$[{\mathrm{CO}}_{2}],RH,\;T,\;t,\;f,\;p_{a}/p_{Cem}$ $,\;p_{a}$ $,\;p_{w}$	19.41	12.01	15.16
4-2	$[{\mathrm{CO}}_{2}],RH,\;T,\;t,\;f,\;p_{a}/p_{Cem}$ $,\;p_{a}$ $,\;p_{FA}$	20.11	13.09	17.01
4-3	$[{\mathrm{CO}}_{2}],RH,\;T,\;t,\;f,\;p_{a}/p_{Cem}$ $,\;p_{a}$ $,\;p_{FA}/p_{B}$	20.89	13.33	16.21
5-1	$[{\mathrm{CO}}_{2}],RH,\;T,\;t,\;f,\;p_{a}/p_{Cem}$ $,\;p_{a}$ $,\;p_{w}$ $,\;p_{FA}$	19.36	12.16	12.77
5-2	$[{\mathrm{CO}}_{2}],RH,\;T,\;t,\;f,\;p_{a}/p_{Cem}$ $,\;p_{a}$ $,\;p_{w}$ $,\;p_{Sd}$	18.70	11.01	14.39
5-3	$[{\mathrm{CO}}_{2}],RH,\;T,\;t,\;p_{Cem}$ $,\;p_{W}$ $,\;p_{FA}$ $,\;p_{G}$ $,\;p_{Sd}$	42.37	17.67	42.27
17-1	[CO_2_], *RH, T, t*, all parameters in Table 2	17.48	11.61	14.05

**Table 5:** Results of different models.

Models	Papadakis [6]		Morinaga [6]		Monteiro [45]		Zhang [46]		Niu [47]		Gong [48]		This Paper	
Error (mm)	AVG ^1^	M ^1^	AVG	M	AVG	M	AVG	M	AVG	M	AVG	M	AVG	M
3 days	15.8	11.3	36.6	30.4	2.7	2.1	3.3	2.7	2.2	1.7	8.2	4.5	2.0	1.4
7 days	22.4	17	55.3	45	4.2	3.3	4.9	4.1	2.6	1.8	12.1	6.7	2.2	1.5
14 days	31.4	22.3	72.5	59.3	6.7	5.3	7.4	6.1	3.4	2.5	16.8	10.1	3.2	2.3
28 days	49.2	35.4	109.5	88.9	9.7	7.7	11.8	10.1	4.4	3.1	27.9	15.6	4.0	2.9
All	37.0	22.8	85.1	58.2	7.7	5.1	8.8	6.0	3.7	2.5	20.6	10.9	3.3	2.3

^1^ AVG denotes the mean value of errors, and M represents the median value of errors.

**Table 6:** Natural carbonation dataset and predicted values [49,50,51,52].

RH (%)	T (°C)	[CO_2_] (%)	*f* (MPa)	*a/c*	*t* (days)	True Values (mm)	Predicted Values (mm)	Errors (mm)
57	13	0.03	38.95	5.85	28	0	0.9	0.9
57	13	0.03	38.95	5.85	56	0	1.3	1.3
57	13	0.03	38.95	5.85	90	1.3	1.6	0.3
57	13	0.03	38.95	5.85	180	7.1	2.3	4.8
57	13	0.03	38.95	5.85	270	9.4	2.8	6.6
57	13	0.03	38.95	5.85	360	9.3	3.3	6.0
63	19	0.03	30.20	6.22	28	1.5	1.7	0.2
63	19	0.03	30.20	6.22	60	2.1	2.5	0.4
63	19	0.03	30.20	6.22	90	2.5	3.0	0.5
63	19	0.03	30.20	6.22	122	2.8	3.5	0.7
63	19	0.03	30.20	6.22	158	3.3	4.0	0.7
63	19	0.03	30.20	6.22	195	3.4	4.5	1.1
63	19	0.03	30.20	6.22	227	3.6	4.8	1.2
63	19	0.03	30.20	6.22	250	3.8	5.1	1.3
63	19	0.03	30.20	6.22	280	4.2	5.4	1.2
63	19	0.03	25.6	6.70	28	1.7	2.1	0.4
63	19	0.03	25.60	6.70	60	2.4	3.1	0.7
63	19	0.03	25.60	6.70	90	2.8	3.8	1.0
63	19	0.03	25.60	6.70	122	3.2	4.4	1.2
63	19	0.03	25.60	6.70	158	3.7	5.0	1.3
63	19	0.03	25.60	6.70	195	3.9	5.5	1.6
63	19	0.03	25.60	6.70	227	4.2	6.0	1.8
63	19	0.03	25.60	6.70	250	4.7	6.3	1.6
63	19	0.03	25.60	6.70	280	5.3	6.6	1.3
63	19	0.03	33.30	6.39	28	2.1	1.5	0.6
63	19	0.03	33.30	6.39	60	2.8	2.2	0.6
63	19	0.03	33.30	6.39	90	3.3	2.7	0.6
63	19	0.03	33.30	6.39	122	3.7	3.1	0.6
63	19	0.03	33.30	6.39	158	4.3	3.5	0.8
63	19	0.03	33.30	6.39	195	4.5	3.9	0.6
63	19	0.03	33.30	6.39	227	4.8	4.2	0.6
63	19	0.03	33.30	6.39	250	5.6	4.5	1.1
63	19	0.03	33.30	6.39	280	6.3	4.7	1.6
63	19	0.03	31.60	7.11	28	2.4	1.6	0.8
63	19	0.03	31.60	7.11	60	3.4	2.4	1.0
63	19	0.03	31.60	7.11	90	4.0	2.9	1.1
63	19	0.03	31.60	7.11	122	4.7	3.4	1.3
63	19	0.03	31.60	7.11	158	5.3	3.9	1.4
63	19	0.03	31.60	7.11	195	5.8	4.3	1.5
63	19	0.03	31.60	7.11	227	5.7	4.7	1.0
63	19	0.03	31.60	7.11	250	7.2	4.9	2.3
63	19	0.03	31.60	8.40	280	7.6	5.3	2.3
63	19	0.03	35.00	8.40	28	2.6	1.5	1.1
63	19	0.03	35.00	8.40	60	3.7	2.1	1.6
63	19	0.03	35.00	8.40	90	4.3	2.6	1.7
63	19	0.03	35.00	8.40	122	4.9	3.1	1.8
63	19	0.03	35.00	8.40	158	5.7	3.5	2.2
63	19	0.03	35.00	8.40	195	6.4	3.9	2.5
63	19	0.03	35.00	8.40	227	6.1	4.2	1.9
63	19	0.03	35.00	8.40	250	7.4	4.4	3.0
63	19	0.03	35.00	8.40	280	8.2	4.6	3.6
63	19	0.03	35.00	9.81	28	3.2	1.5	1.7
63	19	0.03	35.00	9.81	60	4.2	2.2	2.0
63	19	0.03	35.00	9.81	90	4.8	2.7	2.1
63	19	0.03	35.00	9.81	122	5.5	3.2	2.3
63	19	0.03	35.00	9.81	158	6.6	3.6	3.0
63	19	0.03	35.00	9.81	195	7.0	4.0	3.0
63	19	0.03	35.00	9.81	227	7.4	4.3	3.1
63	19	0.03	35.00	9.81	250	8.5	4.5	4.0
63	19	0.03	35.00	9.81	280	9.1	4.8	4.3
57	12.4	0.03	29.80	3.32	2190	5.0	5.7	0.7
57	12.4	0.03	29.80	3.32	9125	15.1	11.6	3.5
75	20	0.03	32.05	4.32	41	1.1	1.3	0.2
75	20	0.03	32.05	4.32	224	2.67	3.0	0.0
73	15	0.03	16.10	6.00	183	6.8	4.9	1.9
73	15	0.03	20.20	6.61	183	5.3	4.0	1.3
73	15	0.03	25.50	6.72	183	4.7	3.1	1.6
73	15	0.03	16.10	6.00	365	7.0	6.9	0.1
73	15	0.03	20.20	6.61	365	6.5	5.7	0.8
73	15	0.03	25.50	6.72	365	4.8	4.4	0.4
73	15	0.03	16.10	6.00	1095	12.1	12.0	0.1
73	15	0.03	20.20	6.61	1095	10.1	9.8	0.3
73	15	0.03	25.50	6.72	1095	9.7	7.6	2.1
73	15	0.03	16.10	6.00	1825	16.1	15.4	0.7
73	15	0.03	20.20	6.61	1825	14.4	12.6	1.8
73	15	0.03	25.50	6.72	1825	9.9	9.9	0.0

## Data Availability

The data presented in this study are available upon request from the corresponding author.
